# The endocannabinoid system: a key modulator of emotions and cognition

**DOI:** 10.3389/fnbeh.2012.00073

**Published:** 2012-11-06

**Authors:** Patrizia Campolongo, Viviana Trezza

**Affiliations:** ^1^Department of Physiology and Pharmacology, University of Rome SapienzaRome, Italy; ^2^Department of Biology, University “Roma Tre”Rome, Italy

The endocannabinoid system is a unique neuromodulatory system in mammalian physiology. It consists of cannabinoid receptors (CB1 and CB2), their endogenous lipid ligands [endocannabinoids, including anandamide (AEA), and 2-arachidonoylglycerol (2-AG)] and the enzymes for ligand synthesis and degradation. In recent years, brain endocannabinoids have emerged as key modulators of affect, motivation and emotions, and the endocannabinoid system is nowadays considered an intriguing target for the development of selective and specific compounds able to treat several psychiatric disorders. This e-book brings together leading experts in the field to provide a deep overview of the physiological and pathophysiological role of the endocannabinoid system in the modulation of emotions and cognition.

The e-book opens with a review where Battista et al. provide a general overview on the endocannabinoid system and then focus on the metabolic and signal transduction pathways of the main endocannabinoids, AEA and 2-AG. At the end, the authors briefly discuss the therapeutic potential of new cannabinoid drugs (Battista et al., 2012). This issue is further elaborated in the following review, where Marco et al. provide both clinical and preclinical evidence supporting the involvement of the endocannabinoid system in several neuropsychiatric disorders (Marco et al., 2011).

The role of the endocannabinoid system in the modulation of emotions and cognition is widely underscored by several reviews of this e-book. Zanettini et al. broadly introduce this topic by discussing the results of studies performed in laboratory animals (Zanettini et al., 2011), while Rubino and Parolaro address this issue from a sexually-dimorphic perspective (Rubino and Parolaro, 2011).

The original research article by Terzian et al. investigated the potential cross-talk between dopaminergic and cannabinoid neurotransmission in the modulation of emotions and cognition (Terzian et al., 2011). The authors showed that conditional CB1 receptor knock-out animals lacking CB1 cannabinoid receptors in neurons expressing D1dopamine receptors exhibited significantly increased contextual and auditory-cued fear compared to wild-type animals, suggesting that a specific reduction of endocannabinoid signaling in neurons expressing dopamine D1 receptor is able to affect acute fear adaptation (Terzian et al., 2011). In their commentary on this research article, Akirav and Fattore discuss about the potential clinical implication of these findings, and indicate the future directions for research in this field (Akirav and Fattore, 2011).

The preclinical studies reviewed by Trezza and co-workers show that cannabinoid modulation of emotionality and cognitive performance appears since early developmental stages; indeed, evidence has been provided over the last few years that animals exposed to cannabinoid drugs during the perinatal, prenatal or adolescent period show long-lasting changes in emotional reactivity and cognitive processing (Campolongo et al., 2007, 2009, 2011; Trezza et al., 2012).

The effects of cannabinoid drugs on hippocampal memory and plasticity are discussed by Akirav (Akirav, 2011); on the basis of the existing literature, she concludes that these effects may vary depending on the route of drug administration, the nature of the task used, whether it involves emotional or non-emotional memory formation, and according to the memory stage under investigation (acquisition, consolidation, retrieval, and extinction) and the brain areas involved (Akirav, 2011).

To study the role of CB1 cannabinoid receptors in the medial prefrontal cortex on cognitive flexibility and emotional behavior in rats, Klugmann et al. upregulated CB1 cannabinoid receptors selectively in this brain area by using adeno-associated viral vector-mediated gene transfer (Klugmann et al., 2011). In their research article, these authors showed that upregulation of CB1 receptors specifically in the rat medial prefrontal cortex induces alterations in emotional reactivity, leads to inadequate social behavior, and impairs cognitive flexibility (Klugmann et al., 2011). In the following research article published on this e-book, Hernandez et al. shed more light on the role of CB1 cannabinoid receptors in mediating reward-seeking behaviors (Hernandez et al., 2011). In particular, the authors showed that, unlike lithium chloride, the CB1 receptor antagonist AM251 did not affect instrumental responding for brain stimulation reward. On the basis of these findings, the authors hypothesize that endocannabinoids are primarily involved with the motivational rather than the intrinsic aspects of reward processing (Hernandez et al., 2011).

The last three articles included in this e-book address the topic of cannabinoid modulation of emotions and cognition from a clinical perspective. The first of these studies is a research article where Spronk and colleagues showed that the active ingredient of Cannabis Δ9-tetrahydrocannabinol (THC) alters performance monitoring, that is a process that allows humans to respond actively and safely to changing environmental demands (Spronk et al., 2011). This study supports the opinion that Cannabis use during performance of complex functions like driving, which require a high level of performance monitoring, might be particularly risky. Fattore and Fratta address a very hot and timely topic, that is the availability of a new generation of drugs that, although devoid of tobacco or Cannabis, when smoked produce effects similar to those induced by THC (Fattore and Fratta, 2011). The authors first outline the general characteristics of these drugs, such as their content and their effects, and then address the consequences that their use has for both health and society (Fattore and Fratta, 2011). The last contribution to this e-book is the opinion article by Bhattacharyya and Sendt, that provides evidence from neuroimaging studies that cannabinoid drugs affect brain areas involved in cognitive and emotional processes (Bhattacharyya and Sendt, 2012).

Altogether, the collection of articles included in this e-book demonstrates that endocannabinoids play a crucial role in the regulation of emotionality and cognitive performance, as outlined by both rodent and human studies. We hope that it will be apparent to the readers how far we have come in recent years in understanding the functions of brain endocannabinoids in both physiological and pathological conditions, and which are the current challenges for researchers working in this field.
